# Educational Mobility and Age-Related Decrements in Kidney Function Across Adulthood Among Black and White Adults

**DOI:** 10.1093/geroni/igab046.1544

**Published:** 2021-12-17

**Authors:** Agus Surachman, Alexis Santos, Jonathan Daw, Lacy Alexander, Christopher Coe, David Almeida

**Affiliations:** 1 University of California San Francisco, San Francisco, California, United States; 2 Pennsylvania State University, University Park, Pennsylvania, United States; 3 The Pennsylvania State University, University Park, Pennsylvania, United States; 4 University of Wisconsin - Madison, Madison, Wisconsin, United States

## Abstract

This paper examines the association between educational mobility and age-related decrements in kidney function. Data from the main survey and the Biomarker Project of the Midlife in the United States (MIDUS) Wave 2 and Refresher samples were combined, resulting in 1,861 adults (54.5% female; age 25-84, Mage=53.37) who self-identified as non-Hispanic Black (n=326) and non-Hispanic white (n=1,535). The estimated glomerular filtration rate (eGFR) was based on serum creatinine, calculated using the CKD-EPI formula. Intergenerational educational mobility was based on the comparison between parental education (no high school/HS degree versus HS degree or higher) and participant’s education level (HS degree or lower versus some college versus bachelor’s degree or higher). Results from regression analysis indicated that Black participants in the moderate upward mobility group (parental education = no HS degree, participant’s education = some college) showed significantly steeper age-related decrements in eGFR across adulthood compared to Black adults with higher stable high status (parental education = HS degree or higher, participant’s education = bachelor’s degree or higher), B=-0.70, SE=0.26, p=.008, or white adults with higher stable high status, B= 0.58, SE=0.29, p=.044. A steeper age-related decrement in eGFR is known as a reliable risk factor for chronic kidney disease and cardiovascular disease. These findings support the notion of skin-deep resilience among Black adults who experience upward socioeconomic mobility. We explored multiple psychosocial factors that may explain these findings, including lifetime and daily discrimination, social status and financial strains, and perceived stress and depressive symptoms.

